# Clinical simulation training improves the clinical performance of Chinese medical students

**DOI:** 10.3402/meo.v20.28796

**Published:** 2015-10-16

**Authors:** Ming-ya Zhang, Xin Cheng, An-ding Xu, Liang-ping Luo, Xuesong Yang

**Affiliations:** 1 Clinical Skills Comprehensive Training Center, The First Affiliated Hospital, Jinan University, Guangzhou, China; 2 Division of Histology and Embryology, Key Laboratory for Regenerative Medicine of the Ministry of Education, Medical College, Jinan University, Guangzhou, China; 3 The First Affiliated Hospital, Jinan University, Guangzhou, China

**Keywords:** medical student, objective structured clinical examination, clinical skill training, simulation

## Abstract

**Background:**

Modern medical education promotes medical students’ clinical operating capacity rather than the mastery of theoretical knowledge. To accomplish this objective, clinical skill training using various simulations was introduced into medical education to cultivate creativity and develop the practical ability of students. However, quantitative analysis of the efficiency of clinical skill training with simulations is lacking.

**Methods:**

In the present study, we compared the mean scores of medical students (Jinan University) who graduated in 2013 and 2014 on 16 stations between traditional training (control) and simulative training groups. In addition, in a clinical skill competition, the objective structured clinical examination (OSCE) scores of participating medical students trained using traditional and simulative training were compared. The data were statistically analyzed and qualitatively described.

**Results:**

The results revealed that simulative training could significantly enhance the graduate score of medical students compared with the control. The OSCE scores of participating medical students in the clinical skill competition, trained using simulations, were dramatically higher than those of students trained through traditional methods, and we also observed that the OSCE marks were significantly increased for the same participant after simulative training for the clinical skill competition.

**Conclusions:**

Taken together, these data indicate that clinical skill training with a variety of simulations could substantially promote the clinical performance of medical students and optimize the resources used for medical education, although a precise analysis of each specialization is needed in the future.

Currently in China, the majority of medical schools use a discipline-based curricular model. Theory, clerkship, and internship are the three main phases. Medical students usually concentrate on the basic science study in the first 2.5–3 years, the length varying according to the school system. Medical students study morphological curricula, then human physiology, biochemistry, immunology, and microbiology before pathology/pathophysiology and pharmacology, etc. Next, they initiate clinical science study in their affiliated hospital as a clerkship, which usually lasts 2 years. Then, medical students acquire clinical skills training as interns for almost 1 year before they graduate.

Clinical skill performance is considered to be a core proficiency and is crucial to professionalism in medical practice, contributing to successful outcomes in patient care. However, because of the lack of patients willing to be examined by medical students, it is hard for medical students to develop the right clinical techniques. Simulation-based medical education has been growing rapidly, becoming one of the most popular teaching methods for improving patient safety and care. The introduction of clinical simulations into traditional medical education dates back to the early 1990s (1, 2). Initially, the simulations principally involved the use of standardized patients or actors to mimic patients with various ailments (3). The standardized patients presented a variety of scenarios for the clinical skills training of medical students; and the application of standardized patients has improved the long-term memory of medical students and the application of their knowledge (4). These innovative simulations are aimed to enhance the patient care and physical examination skills of medical students.

The Clinical Education Center at the First Affiliated Hospital (which is also named Guangzhou Overseas Chinese Hospital), Jinan University, China, was established in September 2009 and further expanded in 2012 into four divisions: a clinical diagnosis laboratory, a surgical laboratory, a medical image laboratory, and a simulative medical education laboratory. The Clinical Skills Comprehensive Training Center was established according to international standards and equipped with a variety of teaching attachments, such as advanced virtual simulations, for clinical skill training in demonstration rooms of different clinical subjects. Thus, this center provides a large variety of medical education programs with simulated clinical encounters to supplement traditional medical education. Now, the center is composed of 16 skills stations and examination rooms with relevant instructors and staff. Each room is equipped with cameras and microphones to record the student–‘patient’ encounters in real time. Multiple platforms have been established, including a common clinical skills training platform, an animal surgical training platform, a medical image training platform, an integrated practice platform for subtropical diseases, an integrated practice platform for common diseases, a first-aid skill training platform, and a clinical skill examination platform. In addition, a sharing platform network was created for medical students to view and emulate new or difficult operative techniques, particularly for operations that are rarely viewed in common situations. The internal network in the center also ensured that medical students could repeatedly view operations anytime and anywhere. In the Clinical Education Center, medical students are trained to perform a comprehensive basic physical examination using a variety of medical practice scenarios; and the training's effectiveness is eventually assessed in examination rooms, reflecting the increasing popularity of the international objective structured clinical examination (OSCE) for assessing the clinical performance of medical students (5, 6).

The objective of the present study is to quantitatively examine the effectiveness of clinical practice training programs that use simulations.

## Methods

### Subjects

The medical students in this study were all recruited from Jinan University and randomly distributed to training hospitals (Guangzhou Overseas Chinese Hospital and non-overseas Chinese hospitals). Medical students doing their clerkship and internship in Guangzhou Overseas Chinese Hospital were selected as the experimental group, and they received both simulative clinical training and traditional medical education training during their clerkship and internship. Medical students doing their clerkship and internship in non-overseas Chinese hospitals were selected as the control group. These students underwent fully traditional medical education training in other hospitals that lacked a simulative clinical skills training center. The traditional medical education system is organized into three parts, consisting of a basic medical science curriculum for the early stage, clinical sciences with intermittent clerkship, and intern practice with clinical exposure. The simulative clinical training is built on the basis of the traditional medical education system, introducing simulative preclinical skills training complemented with real-life clinical skills prior to internship.

In the clinical skills competition, student participants from the 2012-enrolled class were selected as controls because the clinical training center at Guangzhou Overseas Chinese Hospital was not completed when those students practiced in the hospital, whereas the 2013- and 2014-enrolled classes received complete clinical training using simulations.

### OSCE Methods

**Fig. 1:**
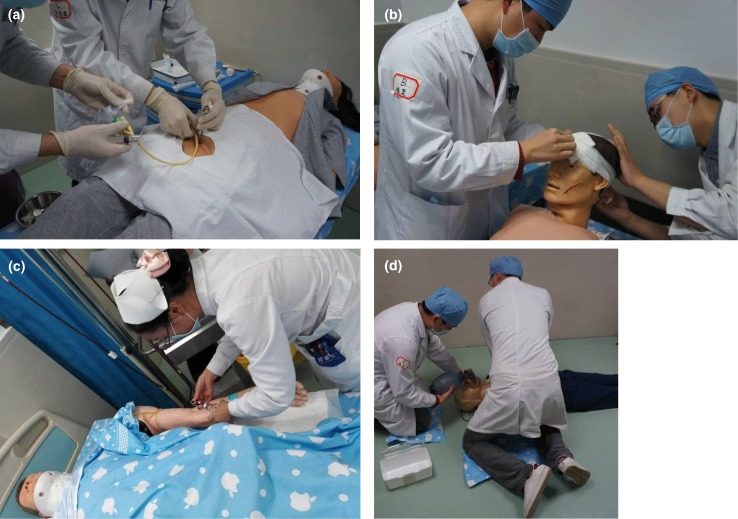
Examples of medical students training with the help of normalized simulations. Images showing examples of the training of medical students using simulations of abdominal puncture (a), wound bandaging (b), cardiopulmonary resuscitation (c), and vacuum blood collection (d).

In 2012, 16 clinical skills training stations were basically completed, including Station 1: Medical Imaging Analysis, Electrocardiogram (ECG) Analysis; Station 2: Cardiopulmonary Resuscitation (CPR); Station 3: Physical Examination; Station 4: Thoracentesis, Lumbar Puncture, Bone Marrow Aspiration, Abdominocentesis; Station 5: Case Analysis of Internal Medicine; Station 6: Auscultation and Palpation on a Simulated Patient; Station 7: Gynecological Examination; Station 8: External Pelvimetry, Four Maneuvers of Leopold; Station 9: History Taking for Internal Medicine; Station 10: History Taking for Obstetrics and Gynecology; Station 11: History Taking for Pediatrics; Station 12: History Taking for Surgery; Station 13: Removing Stitches and Changing Wound Dressing; Station 14: Surgical Hand-washing, Gowning, and Sterile Gloving; Station 15: Preparation of Surgical Site; and Station 16: Making an Incision and Stitches. Eventually, the effects of the clinical skills training with the help of simulations were evaluated using OSCE (7). Several training examples are shown in Fig. 1.

### Ethical considerations

The study was carried out in accordance with the Declaration of Helsinki. The study was not subject to approval bya Research Ethics Committee because no sensitive or personal health information was involved. The medical student participants were provided with written and oral information about the study and informed that they could withdraw at any time. Confidentiality was assured by keeping the materials de-identified in the transcripts, and the data in this study were only accessible to the authors.

### Data analysis

Data analyses and statistical chart preparation were performed using the GraphPad Prism 5 software package (GraphPad Software, San Diego, CA, USA). The results were presented as the means±standard error. One-way ANOVA tests were performed on the data of the OSCE competition scores for the 2012-, 2013-, and 2014-enrolled internists who were simulatively or traditionally trained. A *t*-test was performed on the OSCE competition score data for the 2014 medical students before and after simulative training for 6 months. Other data were analyzed using two-way ANOVA tests. Samples were considered significantly different at *p<*0.05 and extremely significantly different at *p<*0.01.

## Results

### The clinical skill set of medical students trained with simulations was dramatically enhanced

**Fig. 2:**
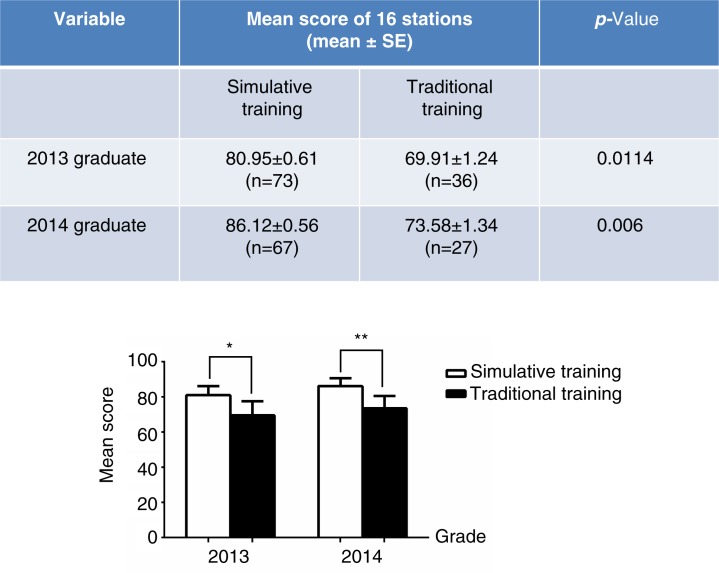
Comparison of the mean scores of 2013- and 2014-graduated interns with simulative versus traditional training. OSCE was carried out among the 2013- and 2014-graduated interns with simulative or traditional training. The upper table shows the mean exam scores (the average score after the students graduated in all 16 stations) of the interns and the *p-*values compared between simulatively and traditionally trained medical students graduating in 2013 and 2014. The lower bar chart shows a comparison of the graduation exam results between simulatively and traditionally trained medical students graduating in 2013 and 2014 (*p<0.05 compared to traditional training, **p<0.01 compared to traditional training).

Among the 109 clinical medicine interns who graduated in 2013, 73 completed clinical practice at Guangzhou Overseas Chinese Hospital of Jinan University and 36 interns practiced at other hospitals. Among all 94 clinical medicine interns who graduated in 2014, 67 completed clinical practice at Guangzhou Overseas Chinese Hospital of Jinan University and 27 practiced at other hospitals. The interns who completed clinical practice at the Overseas Chinese Hospital were fully trained using simulations, whereas the interns at other hospitals did not receive the beneficial clinical training with simulation because of a lack of relevant equipment. To assess the effects of clinical skills training with simulations, we analyzed the exam results of interns trained at Guangzhou Overseas Chinese Hospital and of interns trained at non-overseas Chinese hospitals as a control. The results showed that the mean exam scores in all of 16 stations for both 2013- and 2014-graduated interns who trained at Guangzhou Overseas Chinese Hospital (with simulations) were significantly higher than those of interns trained at non-overseas Chinese hospitals (traditional training, i.e., not trained with simulations; Fig. 2). These results suggest that clinical skills training with a variety of simulations improves the learning ability of medical students.

**Fig. 3:**
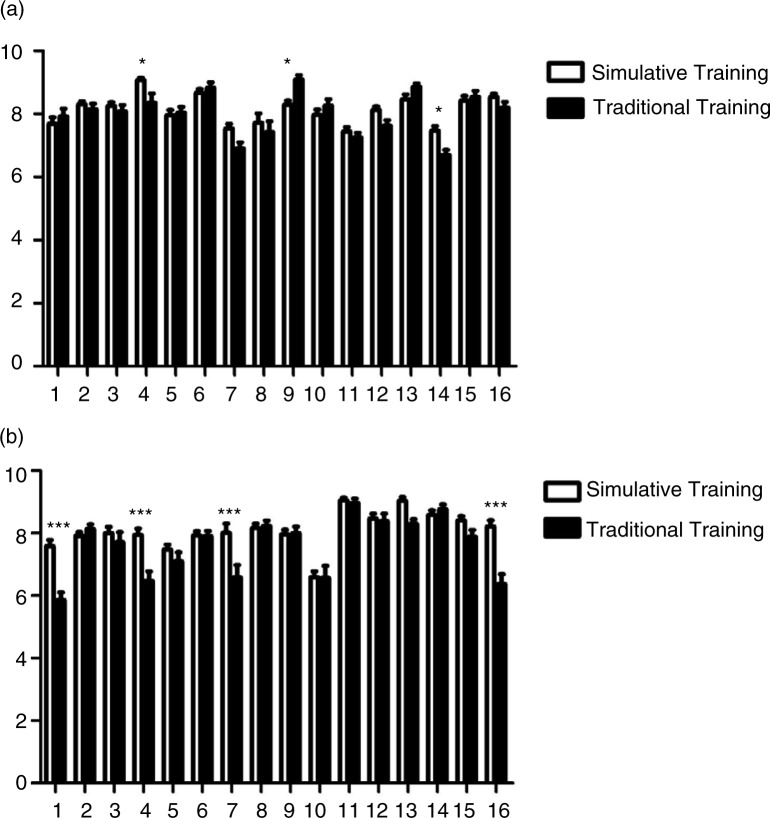
Comparison of the mean scores on 16 clinical skills training stations of 2013- and 2014-graduated interns with simulative versus traditional training. The bar charts show the mean scores on 16 clinical skills training stations between the 2013- (a) and 2014-graduated (b) interns with simulative or traditional training. The exam subjects are indicated as: Station 1: Medical Imaging Analysis, Electrocardiogram Analysis; Station 2: Cardiopulmonary Resuscitation; Station 3: Physical Examination; Station 4: Thoracentesis, Lumbar Puncture, Bone Marrow Aspiration, Abdominocentesis; Station 5: Case Analysis of Internal Medicine; Station 6: Auscultation and Palpation on a Simulated Patient; Station 7: Gynecological Examination; Station 8: External Pelvimetry, Four Maneuvers of Leopold; Station 9: History Taking for Internal Medicine; Station 10: History Taking for Obstetrics and Gynecology; Station 11: History Taking for Pediatrics; Station 12: History Taking for Surgery; Station 13: Removing Stitches and Changing Wound Dressing; Station 14: Surgical Hand-washing, Gowning, and Sterile Gloving; Station 15: Preparation of Surgical Site; and Station 16: Making an Incision and Stitches (*p<0.05 compared to traditional training, ***p<0.00 compared to traditional training).

Moreover, we compared the mean scores on 16 clinical skills training stations of 2013- and 2014-graduated interns comparing simulative versus traditional training. In comparison to traditionally trained students, 2014 graduates who participated in simulative training showed better performance in Medical Imaging Analysis and ECG Analysis, Thoracentesis, Lumbar Puncture, Bone Marrow Aspiration, Abdominocentesis, Gynecological Examination, and Making an Incision and Stitches (Fig. 3b), whereas 2013 graduates showed better performance in Thoracentesis, Lumbar Puncture, Bone Marrow Aspiration, Abdominocentesis, and Removing Stitches and Changing Wound Dressing (Fig. 3a). Meanwhile, students taking traditional training exhibited an advantage over simulatively trained 2013 graduates at collecting medical records (Fig. 3a).

### The training for the clinical skills competition further improved the clinical skill set of medical students

**Fig. 4:**
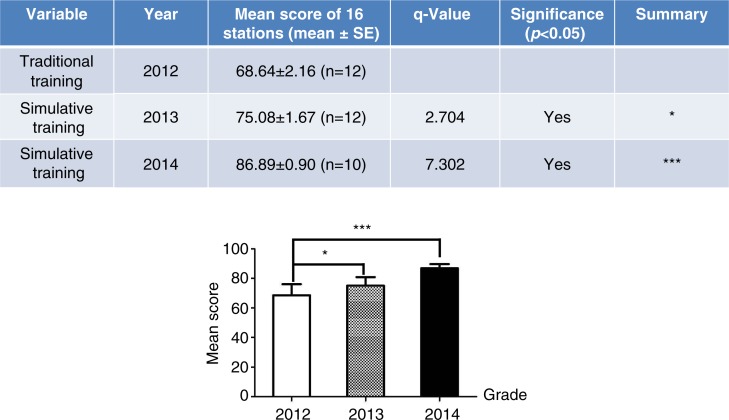
Competition scores were evaluated using OSCE in 2012–2014 graduated interns simulatively or traditionally trained. The upper table shows the mean OSCE scores for performances in all 16 stations among the 2012-graduated medical students (traditionally trained) and the 2013- and 2014-graduated medical students (simulatively trained). The lower bar chart shows a comparison of OSCE scores between simulatively and traditionally trained medical students graduating in 2012 or 2013–2014, respectively (*p<0.05 compared to traditional training, ***p<0.00 compared to traditional training).

To further investigate the effects of clinical skills training with a variety of simulations, we used OSCE to assess the clinical skills mastery of 2012-, 2013-, and 2014-graduated interns (Fig. 4). Note that the 12 medical students who graduated in 2012 did not receive the full clinical skills training with a variety of simulations before participating in the competition, as the simulation training was not yet established. Therefore, the OSCE exam results of the medical students who graduated in 2012 could serve as a control (non-simulation). The training platform was completed in 2012, and as a result, the 12 medical students who graduated in 2013 and the 10 who graduated in 2014 were well trained using a variety of simulations prior to participation in the competition. The table in Fig. 4 shows that the OSCE results for both 2013- and 2014-graduated medical students participating in the competition were higher than those of the medical students graduating in 2012, and statistical significance was observed between both groups (Fig. 4, bar chart).

**Fig. 5:**
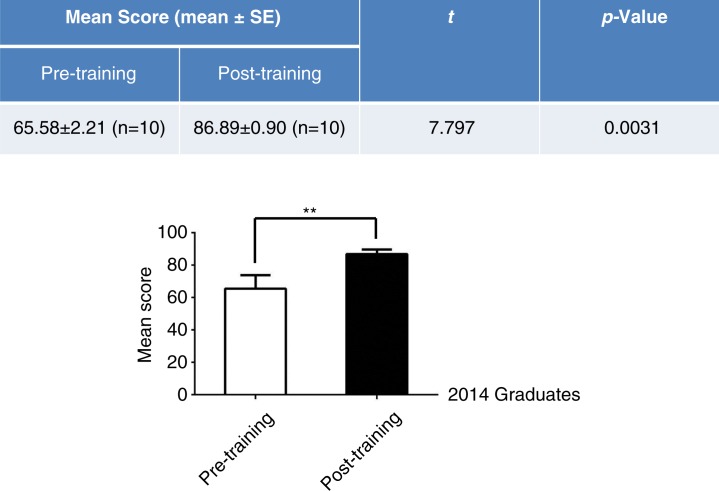
Competition scores were evaluated using OSCE before and after training with simulations. The upper table and lower bar chart show the OSCE results of the 2014 medical students before and after simulative training for 6 months (**p<0.01 compared to pre-training).

To verify these results, we obtained OSCE results from 10 2014-graduated interns before and after training (Fig. 5, table) because these results would reflect the internal effect of clinical skills training with simulations. The OSCE scores of these students were enhanced approximately 20% after training with a variety of simulations (Fig. 5, bar chart). These results indicate that training using simulations was instrumental for students to master clinical skills, indirectly implying the efficiency of clinical skills training using simulations.

## Discussion

Mastering practical clinical skills is absolutely necessary for the training of medical students. However, the concepts and methods of medical education constantly change with time. In traditional medical education, we have generally followed the procedure in which the teachers explain theory in the classroom and demonstrate a variety of practical operations, and subsequently, the medical students practice on patients/manikins. However, traditional teaching methods for training medical students have been challenged recently by the public reaction to medical errors (8). In fact, these problems have existed in traditional medical education for many years. The recent attention to medical errors reflects the increased numbers of enrolled medical students (9). Particularly, medical student enrollment in China has dramatically increased in the last decade, leading to a relative reduction in specialized training time for each medical student and a decrease in hospital resources compared with previous medical education (10). These phenomena are evident not only in China but also in western countries (11–13). Thus, a variety of simulations were introduced into the clinical skill study to assist medical students in mastering clinical skills. The use of simulations solves the problem of poor clinical specialization training resulting from increased enrollment. However, until recently, there has been no quantitative analysis of the effects of simulation on the clinical skill training of medical students in China.

The Clinical Skills Comprehensive Training Center at Guangzhou Overseas Chinese Hospital of Jinan University was endowed and established in 2012 to improve the clinical skills performance of medical students, including a variety of specializations, comprehensive clinical skills, and clinical innovation based on basic clinical skills. At this center, medical students are trained using three-dimensional clinical skills simulations as shown in Fig. 1. Thus, medical students contact ‘patients’ and practice on them early in their training while studying medical theories. The combination of simulation training and clinical practice has greatly remedied the shortcomings of merely teaching theory and the lack of practice patients (14). In addition, the traditional teaching method has been altered to adopt multimedia teaching to presenting content to medical students; thus, clinical medical education more closely resembles actual patient cases with increased authenticity and attractiveness of teaching (15). All alterations of the teaching approach sufficiently mobilize the productive thinking and study interests of medical students and increase the interactivity and communication between teachers and students.

In the present study, we investigated the effect of clinical skills training with simulations on the ability of medical students through a comparative analysis of the graduate exams of traditionally trained and simulatively trained medical students. The diversity of the training approaches reflects historical reasons. Since 2012, as the only hospital directly affiliated with Jinan University, Guangzhou Overseas Chinese Hospital has possessed a clinical skills training platform, but there are no similar facilities at other practice hospitals. The exam results of both 2013- and 2014-enrolled students showed that the medical students trained using simulations at Guangzhou Overseas Chinese Hospital obtained significantly higher scores compared with students trained without simulation at other hospitals (Fig. 2). The effectiveness of clinical skills training using simulations was also assessed through the OSCE clinical skills competition among 2012-, 2013-, and 2014-graduated medical students. To evaluate the training results for these medical students, we used OSCE (7, 16), in which medical students’ ability to apply their knowledge and skills is objectively and professionally evaluated, that is, reducing the subjectivity, occasionality, and variability of traditional exams. The 2012 graduates were used as a control group because the clinical training center was not completely established during the clinical courses in 2011; thus, these students received traditional medical training prior to the competition. However, the 2013 and 2014 graduates adequately utilized the clinical skills training simulations prior to the competition. A comparison of the results from the OSCE showed that the 2013 and 2014 medical students had significantly higher OSCE scores in the clinical skills competition compared with the 2012 students (Fig. 4). This result was confirmed through a comparison of OSCE results prior to training with those obtained after training for the 2014 students. Indeed, the OSCE results were dramatically enhanced after the clinical skills training using simulations (Fig. 5), further suggesting that clinical skills training using simulations could effectively improve the clinical performance of medical students.

The content of 16 skill simulative training stations was arranged according to mature courses developed by other medical schools with a modification based on our own current conditions. To coordinate clinical teaching, this center utilizes various medical simulations with medical equipment and models, including defibrillators, ECG monitors, liver–spleen palpation models, cardiopulmonary auscultation models, CPR models, etc. The results from the quantitative analysis of the mean scores on 16 clinical skills training stations were organized into two performance domains: clinical operating skills and clinical reasoning skills. As shown by the results, simulative training enhances the capability of the students in practical clinical skills, such as thoracentesis, lumbar puncture, bone marrow aspiration, abdominocentesis, physical examination, and surgical skills. Meanwhile, we also found that there was no significant difference in medical history taking between simulatively trained and traditionally trained students, the latter even sometimes being superior, which implies that simulation did not exhibit an impact on clinical reasoning skills. These results imply that the content of simulative training should be adjusted according to the analysis of actual effects.

## Conclusions

In summary, the quantitative data showed that the application of clinical skills training using a variety of simulations improved clinical medical education, particularly the clinical skills training of medical students, thereby promoting the integrative abilities of medical students. Notably, the effectiveness of simulative training for each specialization was not analyzed and should be addressed in the future.
